# Switchable and Tunable Terahertz Metamaterial Absorber with Ultra-Broadband and Multi-Band Response for Cancer Detection

**DOI:** 10.3390/s25051463

**Published:** 2025-02-27

**Authors:** Yadgar I. Abdulkarim

**Affiliations:** 1 Regional Center of Advanced Technologies and Materials, Czech Advanced Technology and Research Institute (CATRIN), Palacký University Olomouc, 779 00 Olomouc, Czech Republic; yadgar.abdulkarim@upol.cz; 2 Physics Department, College of Science, Charmo University, Kurdistan Region, Chamchamal 46023, Iraq

**Keywords:** metamaterials THz absorber, vanadium dioxide, graphene, polarization angle, incident angle, cancer detection, Multifunctional devices

## Abstract

This paper proposes a switchable and tunable terahertz metamaterial absorber utilizing a graphene-VO_2_ layered structure. The design employs reconfigurable seven-layer architecture from top to bottom as (topaz/VO_2_/topaz/Si/graphene/topaz/Au). CST software 2018 was used to simulate the absorption properties of terahertz waves (0–14 THz). The proposed metamaterial exhibits dual functionalities depending on the VO_2_ phase state. In the insulating state, the design achieves a tri-band response with distinct peaks at 3.12 THz, 5.65 THz, and 7.24 THz. Conversely, the VO_2_’s conducting state enables ultra-broadband absorption from 2.52 THz to 11.62 THz. Extensive simulations were conducted to demonstrate the tunability of absorption: Simulated absorption spectra were obtained for broadband and multi-band states. Electric field distributions were analyzed at resonance frequencies for both conducting and insulating states. The impact was studied of VO_2_ conductivity, loss tangent, and graphene’s chemical potential on absorption. The influence was investigated of topaz layer thickness on the absorption spectrum. Absorption behavior was examined of VO_2_ under different states and layer configurations. Variations were analyzed of absorption spectra with frequency, polarization angle, and incident angle. The proposed design used for the detection of cervical and breast cancer detection and the sensitivity is about is 0.2489 THz/RIU. The proposed design holds significant promise for real-world applications due to its reconfigurability. This tunability allows for tailoring absorption properties across a broad terahertz range, making it suitable for advanced devices like filters, modulators, and perfect absorbers.

## 1. Introduction

The terahertz (THz) frequency band (frequencies from 0.1–10 THz) is an exciting, largely unexplored portion of the electromagnetic spectrum with numerous applications spanning security screening [1], medical imaging [2], and communication technologies [3]. Nonetheless, facile control of THz waves through absorption is still difficult because few can be effective materials for absorption. This is where metamaterials came into plays, which are artificial materials with properties that are not found in nature. Metamaterials can be used in various applications ranging from medical application [4,5,6], Antenna [7,8,9,10], clocking [11,12], sensing [13,14] and perfect absorber [15,16,17]. Because of these properties scientists can arrange their structure very deliberately, making them ideal metamaterials for interacting with THz waves.

THz metamaterial absorbers exhibit unique properties such as broadband absorption (unlike traditional materials limited to specific frequencies) and tunable absorption (adjustable via external factors like temperature or light) [18,19,20]. Their nanostructured design enables THz miniaturization, surpassing conventional technologies [21] These features make them ideal for applications like THz shielding (protecting electronics from radiation), THz imaging (enhancing medical/security cameras), and metamaterial-based modulators for 5 G/THz communication [22,23,24]. Ongoing research promises further advancements, enabling customizable and emerging THz applications.

Graphene, a 2D honeycomb lattice of carbon atoms, excels in terahertz-wave manipulation due to its electrical and optical properties [25]. Its high conductivity enables strong interaction with terahertz radiation. By adjusting conductivity via voltage or chemical doping, graphene’s absorption features can be dynamically controlled. It supports surface plasmons—collective electron oscillations at its surface—which enhance terahertz wave absorption. This allows switchable and tunable absorption in metamaterial design [26,27].

VO_2_ (Vanadium Dioxide) is critical for enabling specific functionalities in meatamatrial/metasurface absorbers at terahertz (THz) frequencies [28]. Its phase transition from an insulating to a conducting state allows thermal control of absorptance, enabling ultra-broadband absorption in the conductive state and multi-band absorption in the insulating state [29,30].

Both graphene and VO_2_ benefit the metamaterial design greatly when combined: Chengwei Song et al. and Pengyu Zhang et al. [31,32] usefully designed ultra-broadband THz metamaterials absorber: The absorption of THz waves can be efficiently carried out in the conducting state of VO_2_. S. Zhuo et al. and Dan Hu independently developed a new THz metamaterials broadband and dual-channel perfect absorbers based Graphene and VO_2_ layers [33,34]. With the help of graphene, over an extremely broad frequency range Metamaterials can achieved Multi-band Absorption [30]: In the insulating state of VO_2_, the metamaterial displays more bands absorption originating from the interaction of graphene conductivity and properties of VO_2_ layer. This results in focused absorption at desired THz frequencies. Another benefit is Reconfigurability—The ability to transition between the insulating and conducting states of VO_2_ with stimulus enables dynamic manipulation of the absorption response from the metamaterial [35]. These results provide many possibilities to new THz devices with controllable functions.

In recent years, biomedical researchers have developed systems for detecting cervical and breast cancer cells by analyzing dielectric property differences between normal and cancerous cells. For instance, this approach is discussed in detail in [36].

In this work, we propose a switchable and tunable VO_2_ phase transition terahertz metamaterial absorber. The absorber toggles between ultra-broadband (metallic VO_2_ state) and tri-band absorption (insulating VO_2_ state) via external stimuli. It also exhibits high sensitivity for detecting cancers such as cervical and breast cancer.

## 2. Exploring the Reconfigurable Metamaterial Design

**Figure 1:**
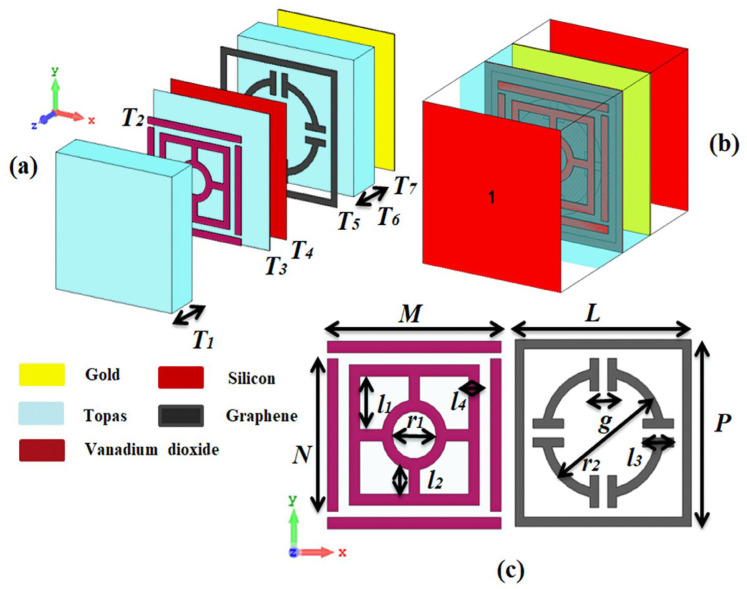
(**a**) Perspective view of the proposed metamaterial structure, showing the seven-layer stack. (**b**) Numerical setup in CST simulation 2018 software, depicting the two ports used for excitation and measurement. (**c**) Front view of the VO_2_ layer (left) and graphene layer (right), highlighting their respective configurations within the structure.

The seven-layered structure of the proposed metamaterial absorber provides a significant potential for tunable and switchable terahertz wave absorption as shown in Figure 1a. The individual layer and understand their role in the overall working is explained from the top layer to bottom as below: 1. Topaz 1: This dielectric spacer material is included in the metamaterial. The dielectric spacers are to keep the distance between the metallic layers and significantly influence the interaction of light waves with the structure. This is in contrast to using standard polymers in metamaterial designs, Topaz as dielectric can be set to its well-known dielectric properties thereby allowing the controlled propagation of terahertz waves in the metamaterial in this design the dielectric constant of topaz was set 2.35. 2. Vanadium Dioxide (VO_2_)—An Unique Layer for Reconfigurability The insulator-metal phase transition of VO_2_ occurring at a critical temperature (about 68 °C). In its insulating form, VO_2_ is essentially transparent to terahertz waves. Otherwise, in its metallic state, VO_2_ obstructs wave propagation enough to provide significantly better absorption. This is a temperature-dependent physical property which makes it easy to control absorption behavior of metamaterial. In our design a conducting (metallic) state of VO_2_ was fixed with the conductivity of 3 × 10^5^ S/m. However, in the insulating state, when the conductivity is reduced to 3 × 10^−5^ S/m. 3. Topaz (third layer)—another dielectric spacer, this layer tunes the terahertz-wave interaction with the VO_2_ layer and the substrate metallic components more. 4. This layer does double duty. Being a dielectric substrate firstly but also on which the metamaterial is built up on and physically supported. Second, silicon provides a means for patterning additional resonances into the metamaterial design, which potentially enables sharper absorption peaks or the ability to tailor absorption characteristics, the dielectric constant of Si is 3.5. Graphene plays a critical role for enabling ultra-broadband absorption. Because of its high conductivity and novel relationship with the terahertz waves, graphene plays a crucial role in the total absorption efficiency of the device. A more aggressive patterning or design of the graphene layer could additionally optimize the strategic absorption of the design characteristics. In this work inside the numerical software we utilize the Fermi energy level of the graphene layer was set to be 0.9 eV and the time relaxation for broadband fixed on 0.1 ps, while for the Multiband determined to 1 ps, [26]. 6. Topaz 2: This final dielectric also blocks the air layer from the metamaterial, where layer is ensures best wave transmission from air layer into metamaterial core. 7. Gold: The lowest stratum is a metal ground plane with electrical conductivity of 4.561 × 10^7^ S/m. The metamaterial cavity comprises layers 2, 3, and 4, and the layer 4 of this structure can block all the terahertz waves that manage to penetrate the previous layers, meaning full absorption in the metamaterial cavity.

In order to develop the new design, the absorptive properties of terahertz waves for the proposed absorber were carefully simulated using CST Studio Suite, 2018 software for electrical engineers to simulate electromagnetic waves on different kinds of materials and structures. These simulations were performed at frequencies spanning from 0 to 14 THz, yielding critical information regarding the absorption behaviors of the metamaterial. This data can be used to further tailor the specific target absorption features. By understanding the role of the different layers and how they interact, we gain valuable insights into the potential of this reconfigurable metamaterial design for terahertz wave absorbers. In this work, unit cell boundary conditions were applied on both the X and Y axes, while the Z-axis was set to open add space. The Kubo formula connects a material’s response to an applied electric field (model the graphene conductivity) with the material’s equilibrium properties using the following equation [37,38]:

(1) $$\sigma \left(\omega .\mu_{c}.\Gamma .T\right)=\sigma_{inter}+\sigma_{intra}$$

where σ_inter and σ_intra are represented interband conductivity contribution and intraband contribution respectively. The integral conductivity can be written by this equation, while the intra-band conductivity represented by Equation (3)

(2) $$\sigma_{inter}=\frac{ie^{2}}{4\hbar }ln\left[\frac{2\left|\mu_{c}\right|-(\omega +i\tau^{-1})\hbar }{2\left|\mu_{c}\right|+(\omega +i\tau^{-1})\hbar }\right]$$

(3) $$\sigma_{intra}=\frac{ie^{2}k_{B}T}{\pi h^{2}(\omega +i\tau^{-1})}\left[\frac{\mu_{c}}{k_{B}T}+2\ln(e^{\frac{\mu_{c}}{k_{B}T}}+1\right]$$

All symbols in Equations (2) and (3) are defined by the following: e = the electrical charge, ω = angular frequency, *μc* = chemical potential of graphene, *T* = is the temperature, *τ* = relaxation time, kB is the Boltzmann constant and finally *ħ* = Planck’s constant. In the Equation (3) When *kBT* << *μc*, the chemical potential equals the Fermi energy level. Applying a voltage to a gate electrode deposited near the graphene layer electrostatically modifies the charge carrier concentration and, consequently, the Fermi level. And chemical doping are the two practical techniques to achieving Fermi energy level of graphene. The chemical potential of graphene can found by using this equation [39]

(4) $$\mu_{c}=\hbar \upsilon_{f}\sqrt{\pi a_{0}V_{bias}}$$

In the above equation the parameters are defined by *ħ* is the Planck’s constant, *vf* is Fermi velocity, *α*_0_ is a constant, is the bias voltage. The simulation based on goes with CST Microwave Studio 2018 commercial software and simulates a material used in optical applications which is graphene. This gives the possibility to tune the absorbance of the proposed structure dynamically by varying the Fermi energy level of the graphene. Table 1 show the optimized parameters used in numerical CST 2018 software.

The permittivity of the dielectric layer VO_2_ is modeled by the Drude model and described by [27].

(5) $$\varepsilon (\omega )=\varepsilon_{\infty -}\frac{\omega_{p}^{2}(\sigma )}{\omega^{2}+i\gamma \omega }$$

where $\omega_{p}^{2}(\sigma )$, γ, ω, and $\varepsilon_{\infty }$ plasma frequency depends on conductivity, collision frequency which is equal to 5.75 × 10^13^ rad/s, angular frequency, and electrical permittivity at infinite respectively.

Plasma frequency which is defined by

(6) $$\omega_{p}^{2}\left(\sigma \right)=\frac{\sigma }{\sigma_{0}}\omega_{p}^{2}(\sigma_{0})$$

where σ_0_ = 3 × 10^5^ S/m and *ω_p_*(*σ*_0_) = 1.5 × 10^15^ rad/s.

In our design a conducting (metallic) state of VO_2_ was fixed with the conductivity of 3 × 10^5^ S/m. The electrical (σ), thermal (κT) and optical (ϵ) actions can modify the conductivity properties of a material, introducing new values of the plasma frequency (ωp(σ)).

## 3. Simulated Results and Discussion: Switchable and Tunable Terahertz Metamaterial Absorber

**Figure 2:**
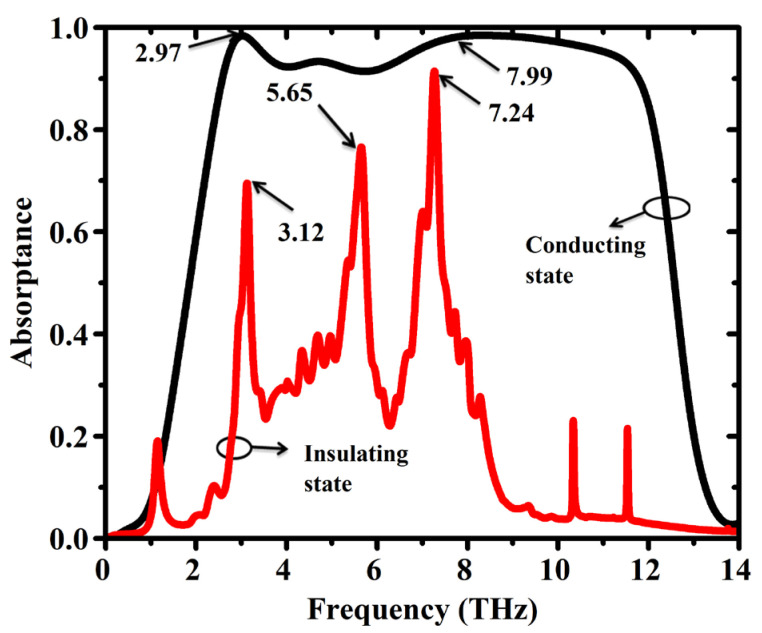
Absorption spectra of the proposed metamaterial containing VO_2_ in different conduction states, calculated in a simulation. Black solid line: VO_2_ conducts; red solid line: VO_2_ insulates.

As a proof of concept of the switchable THz metamaterial absorbers, the simulated results are presented in Figure 2. Here we show the simulated absorption characteristics of our novel reconfigurable graphene-VO_2_ terahertz metamaterial absorbance design. The influence of the VO_2_ phase transition on the absorption behavior was studied. The CST 2018 software was used to simulate the metamaterial structure. Unit cell boundaries were then imposed in the x and y directions to model the layers of the metamaterial periodically. An open space boundary condition was used along the z-axis to simulate the free space propagation of terahertz waves. We consider the VO_2_ layer in two states: insulating and conducting. The material properties for each state were relayed to the simulation mode, The S_11_ and S_21_ (reflection and transmission) parameters for both VO_2_ states have been obtained from these simulations. From S_11_ and S_21_ parameters, absorption coefficient (A) of each VO_2_ state was calculated using the following equation:

A = 1 − |S_11_|^^2^ − |S_21_|^^2^ (7)

VO_2_ state (Black Solid Line) Simulation Results indicate the terahertz ultra-broadband absorption response is significantly achieved. The measured absorption is above 90% in a broad frequency window ranging from 2.56 THz to 11.82 THz. This proves that such kind of resonant nature of this metamaterial design is capable of absorbing terahertz wave efficiently with respect to the microwave conducting state of VO_2_. The term “resonant nature” refers to the collective electromagnetic response of the metamaterial structure, which arises from the interaction of the patterned layers (VO_2_ and graphene) with the incident terahertz waves. While the absorption is broadband, it is achieved through the superposition of multiple resonances and impedance matching effects enabled by the design. These resonances are not narrowband but rather contribute collectively to the wideband absorption behavior.

The simulative results demonstrate the effectiveness of this design for a switchable and tunable THz metamaterial absorber. As such, the ability to control the phase transition of VO_2_ leads to divergent absorption characteristics, which allows for a broad range of functionality. The high absorption observed in the conducting VO_2_ state implies potential applications in terahertz wave shielding, energy harvesting, and thermal management in the terahertz regime.

In the insulating state, VO_2_ absorption (red solid line) shows multi-band absorption, contrasting the broad current-response-generating absorption in the conducting state. There are three separate resonance peaks at 3.12 THz, 5.65 THz, and 7.24 THz frequencies. These spectral responses essentially imply the tunable absorptivity of the metamaterial, enabled by the switching capabilities of VO_2_ to absorb terahertz waves at selected frequencies in the insulating state. The resonance peaks for the insulating VO_2_ state suggest the possibility of narrowband terahertz filters or sensors that can be dynamically tuned by adjusting the VO_2_ temperature.

**Figure 3:**
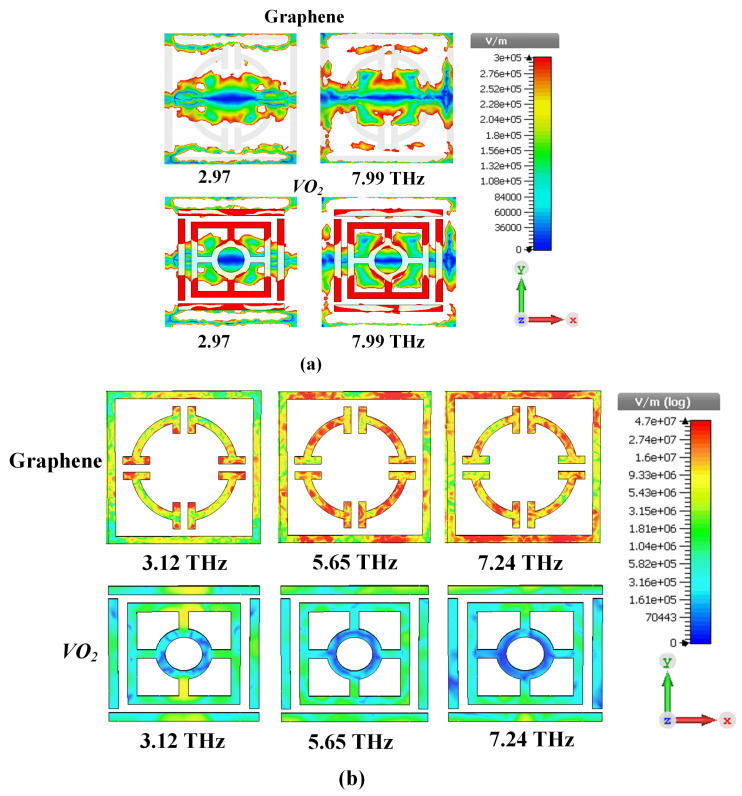
Color map simulated electric-field simulation of *TE* (transverse electric) polarized light at normal incidence 0.7 eV chemical potential (μc) of the front views are illustrated (**a**) broader absorber: Electric field distributions at about 2.97 THz and 7.99 THz. (**b**) presents the simulated electric field distributions at 3.12, 5.65 and 7.24 THz of the multi-band absorber, for both graphene and VO_2_ respectively.

This figure (Color map simulated electric-field simulation of *TE* polarized light at normal incidence 0.7 eV chemical potential (μc) of the top view) demonstrates how the Electric field (E-field) interacts with the proposed metamaterial absorption structure under TE polarization (E-field oscillates perpendicular to the direction of light propagation) with normal incidence (Light hits the surface head-on). 0.7 eV is the chemical potential (*μc*) related to a specific state in the material that significantly affects the electrical properties of it. Figure 3a: Shows a lower resonance frequency (2.97 THz) where the E-field is localized in both the graphene layer and the VO_2_ ring resonator which is at the edge of both layers. Because it does, it means that the light couples most effectively to this portion of the structure at this frequency. In this frequency (7.99 THz), which is the second resonance frequency, the E-field inside the outer resonator becomes stronger comparing to first resonator frequency in both VO_2_ and graphene layers. This indicates a change in the field-forming pattern with a more localized E-field occurring at the outer ring top.

Figure 3b shows the resonance frequencies of 3.12 THz, 5.65 THz, and 7.24 THz, the electric field distribution is expected to exhibit strong localization and enhancement. This is because resonance occurs when the incident electromagnetic waves couple strongly with the structure, leading to a buildup of the electric field in specific regions.

Regarding the field distributions of graphene and VO_2_. We confirm that they are not the same at different frequencies. This variation arises due to the frequency-dependent response of the materials. Graphene and VO_2_ exhibit distinct electromagnetic properties at different frequencies, which influence their interaction with the incident terahertz waves. For instance, graphene’s conductivity and VO_2_’s phase transition (from insulating to metallic state) are highly sensitive to frequency changes, leading to variations in field distributions. These differences are critical for achieving the tunable and switchable functionalities of the proposed metamaterial absorber.

In summary, the electric field distribution patterns expose how the design influences the light-metamaterial interaction, culminating in frequency-specific absorption effects. By tuning the frequency, the researchers can dictate where within the structure light interacts most intensely, leading to broadband or multiband properties.

The proposed structure is designed to achieve tunable absorption through the synergistic interaction of its constituent materials. Below, we explain the role of each layer and the mechanisms governing their interactions: The graphene layer plays a critical role in enhancing absorption due to its tunable conductivity and strong interaction with terahertz waves. When the graphene layer is removed, the absorption curve shows a significant reduction in performance, particularly in the multi-band regions. This is because graphene’s surface plasmon resonance and its ability to confine electric fields are essential for achieving high absorption at specific frequencies. The absence of graphene disrupts this resonance, leading to lower absorption efficiency.

The VO_2_ layer contributes to the tunability and switchability of the absorber due to its phase transition properties (insulating to metallic state). When the VO_2_ layer is removed, the absorber loses its ability to dynamically adjust its absorption properties. The absence of VO_2_ also affects the impedance matching of the structure, leading to a mismatch between the metamaterial and free space, which reduces absorption across the spectrum.

The dielectric spacer is crucial for maintaining the proper distance between the graphene and VO_2_ layers, enabling constructive interference and optimal coupling of electromagnetic waves. When the dielectric spacer is removed, the absorption curve shows a broad reduction in performance, as the interference conditions are no longer met. The spacer also helps in impedance matching, and its absence leads to increased reflection and reduced absorption.

The high absorption performance of the proposed metamaterial absorber arises from the synergistic interaction between the graphene, VO_2_, and dielectric spacer layers. Graphene provides strong field confinement and plasmonic effects, while VO_2_ enables dynamic tunability. The dielectric spacer ensures proper phase matching and impedance matching, allowing the structure to achieve ultra-broadband and multi-band absorption. The interplay between these layers creates a highly efficient and reconfigurable absorber.

**Figure 4:**
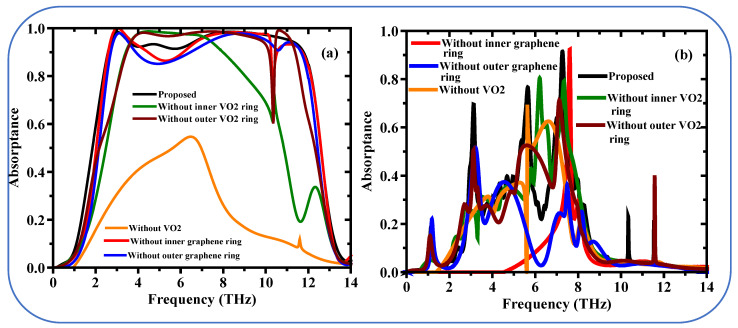
There are absorption spectrums made numerically on different structures (**a**) broadband, (**b**) multi-band.

Figure 4a investigates the effect of different layers on the absorption of the proposed metamaterial design in conducting state (black line) In this study, we examine the absorption response throughout the frequency range between 0 to 14 THz. Proposed Structure (Black Line): The complete metamaterial design has a ultra-broadband absorption response, which is shown by the black line. When structured as such, this absorption extends above 90% over a broad frequency range of about 2.56 THz to 11.82 THz. Impact of VO_2_ Rings resonators (Green and Wine Lines): The green line shows the absorption response when the inner VO_2_ ring is absent. This manipulation yields a broadband which ranges from only 3.4 THz to 7.99 THz, which is much more limited compared to the full design. The Region of the VO_2_ Ring resonator, excluding the outer VO_2_ ring, is given to the structure as a wine line. A larger absorption band is obtained in a good agreement with an average absorption 90% over a larger band of frequency (3.47 THz to 10.12 THz). The absorption that would be obtained if the entire VO_2_ layer were not included (Orange Line). One resonant peak is found around 6.49 THz with an absorption value less than 60% for this structure, which demonstrates the necessity of VO_2_ layer in realizing broadband absorption. Effect of Graphene Rings resonator (Red and Blue Lines): The red line indicates designs without the inner graphene ring. It preserves an exceptionally wide absorption band ranging from 2.59 THz to 10.12 THz with absorption efficiency approximately 90%, but it also creates several dips in the band where the absorption is below 90%. The inner graphene ring makes a uniform high absorption over the bandwidth finally; the blue line represents the structure without the outer graphene ring resonator. This configuration enables a more broadband response to the proposed design (2.69–11.7 THz). It has reduced absorptions between 3.96 THz to 6.24 THz below 90%, but it is huge in the whole range with absorptions close to 100%. In conclusion we show that the variation of all these layers proposed design resulted in significant broadband absorption performance compared to what has been explored here. The absorption exceeds 90% across most of the bandwidth thus also can be employed as the absorber for the widest operation range (2.56 THz to 11.82 THz). Remarkably, these results highlighted the importance of individual layer to the total performance of the metamaterial absorber.

Figure 4b Image of a multi-layer depicted proposed structure (full structure) which appears to entail stuff like VO_2_ resonators and graphene rings. The total three resonance peaks indicated by the (black line on the Figure 4b) are excited at particular terahertz frequencies (3.12 THz, 5.65 THz, 7.24 THz) and have good absorption. By removing different layers on the proposed structure and keep other fixed the absorption profile significantly changed. If remove both the inner and outer VO_2_ resonators independently, then two small peaks appear as shown by a (Green Line) and (Wine Line), which is clearly not a practical application. Without the VO_2_ layer completely (Orange Line), there is a single absorption peak at about 5.6 THz at an absorptivity of 70% yet this may not be optimum. One strong peak due to the inner graphene ring (Red Line) absence. Single weak peak remains when the outer graphene ring resonator (blue line) is removed.

In summary the full structure proposed would potentially show a multiple band response which can be useful in terahertz application. All the layers contribute in the absorption profiles which are VO_2_ resonators and graphene rings.

**Figure 5:**
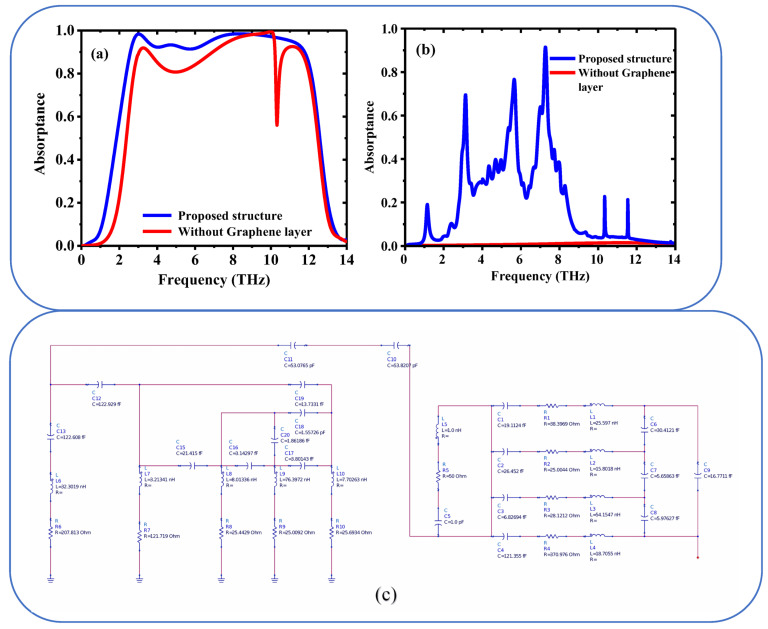
Simulated proposed structure with and without Graphene layer (**a**) Conducting state, (**b**) Insulating state and (**c**) Equivalent circuit diagram for the proposed THz metamaterials absorber.

Figure 5a,b depict the simulated proposed structure with and without a Graphene layer, showing two different states:

The Figure 5a illustrate the structure when it is in a conducting state. The presence of the Graphene layer likely enhances the conductivity, allowing for efficient electron flow through the material. The Graphene layer may act as a conductive pathway, enabling the structure to function as a conductor. Figure 5b: shows the structure in an insulating state, where the Graphene layer is either absent or not contributing to conductivity. In this state, the material prevents electron flow, acting as an insulator. The absence or non-functionality of the Graphene layer results in a lack of conductive pathways, making the structure resistant to electrical current.

Figure 5c shows the ultra-broad absorption mechanism in the proposed seven-layer design (Topaz (*T*_1_)—VO_2_ ring (*T*_2_)—thin Topaz (*T*_3_)—Silicon (*T*_4_)—Graphene ring (*T*_5_)—Topaz (*T*_6_)—Gold (*T*_7_)) can be effectively explained using an equivalent circuit model [40]. This model represents the electromagnetic interactions and material properties of each individual layer. The mechanism hinges on achieving broadband impedance matching to minimize reflection and maximize absorption across a wide frequency range.

Topaz (*T*_1_): Acts as a dielectric layer. It can be represented by a capacitor *C*_1_ in the equivalent circuit. VO_2_ ring (*T*_2_): VO_2_ is a phase-change material that can switch between insulating and metallic states. It can be modeled as a variable resistor *R*_1_ and inductor *L*_1_ in series, depending on its phase state. Thin Topaz (*T*_3_): Another dielectric layer, represented by a capacitor *C*_2_. Silicon (*T*_4_): Silicon is a semiconductor and can be modeled as a resistor *R*_2_ and capacitor *C*_3_ in parallel. Graphene ring (*T*_5_): Graphene is a highly conductive material with tunable properties. It can be represented by a resistor *R*_3_ and inductor *L*_2_ in series. Topaz (*T*_6_): Another dielectric layer, represented by a capacitor *C*_4_. Gold (*T*_7_): Gold is a highly conductive metal and can be modeled as a resistor *R*_4_ and inductor *L*_3_ in series. If the components are connected in series, the total impedance $Z_{total}$ of the circuit is the sum of the impedances of all the components.

The resonance frequency *f* is determined by the condition that the imaginary part of the total impedance is zero, i.e., $Im(Z_{total}=0)$.

If the inductors L_1_, L_2_, L_3_, …, C_1_ + C_2_ + C_3_ + and R_1_ + R_2_ + R_3_ + are connected in series, the total inductance, capacitance and resistance are L_total_ = L_1_ + L_2_ + L_3_ +, the C_total_ = C_1_ + C_2_ + C_3_ + and R_totsl_ = R_1_ + R_2_ + R_3_ +.

In the proposed design the structure consists of resistance, inductance and capacitance therefor can be represented by *RLC* as below,

(8) $$Z_{total}=R_{total}+j\omega L_{total}+\frac{1}{j\omega \;C_{total}}$$

where *ω* = 2πf is the angular frequency

Resonance occurs when the imaginary part of the total impedance is zero:

(9) $$Im\left(Z_{total}\right)=\omega L_{total}-\frac{1}{\omega \;C_{total}}=0$$

Solving for *ω*

(10) $$\omega^{2}=\frac{1}{L_{total}\;C_{total}}$$

Putting the Equation (3) in *ω* = 2 πf

Thus, the resonance frequency

(11) $$f=\frac{1}{2\pi \sqrt{L_{total}\;C_{total}}}$$

The equivalent circuit diagram was designed and simulated using ADS (Advanced Design System) and we calculated the resonance frequency to be 0.1166 THz. The actual resonance frequency depends on the specific values of *L* and *C.*

**Figure 6:**
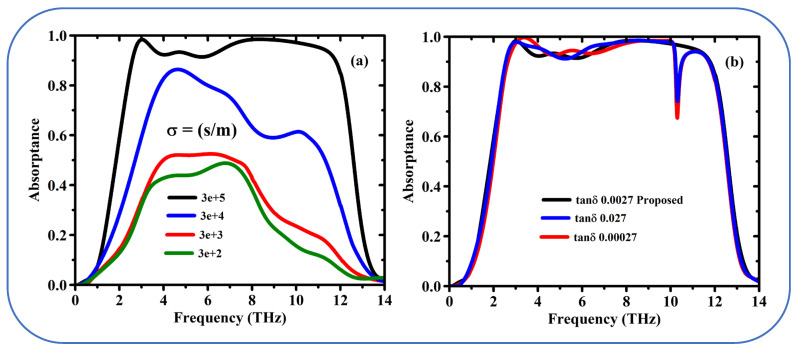
Simulated absorption responses of the proposed design (**a**) for different conductivities of VO_2_ and (**b**) for various loss tangents.

Figure 6a illustrates the variation in conductivity of VO_2_ in its conducting state, where the suggested design (black line) represents the ideal metamaterial structure with ultra-broad absorption across a wide frequency range: It is possible that the black line represents a wide frequency range in which the material is absorbing light greatly. In study mentions that as conductivity of the conducting the VO_2_ decreases, the rate of absorption also decreases. This shows that the absorption of VO_2_ is strongly influenced by its conductivity, which led us to explore the realization of a tunable metamaterial. Figure 6b, shows the impact of modulating the loss tangent of VO_2_ on the absorption. The Figure 6b refers to three values: 0.0027, 0.027, and 0.00027. But changing the loss tangent within the given range, i.e., among the three values arouses minimal impact on the absorption, save for a single frequency (approximately at 10.2 THz) for which the absorption weakens for the blue and red curves.

In summary, Figure 6 probably reveals the principle that the VO_2_-supported metamaterial really exhibits an encompassing light absorption band. The absorption properties are tunable by manipulating the conductivity of VO_2_, with change in loss tangent over a certain range that seems to have little effect on wave absorption.

**Figure 7:**
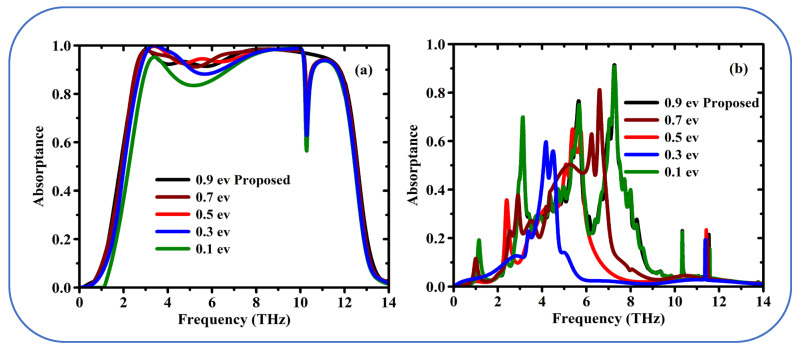
Simulated absorption spectrum as a function of the frequency by changing chemical potential of graphene depending on the state of VO_2_. (**a**) Metallic state, and (**b**) Insulating state.

A single layer of carbon atoms with exceptional electrical properties. Conductivity of graphene is tunable as a function of the chemical potential (Fermi energy level, *μc*). The Figure 7a depicts the absorption process of VO_2_ when it is in a metallic state for different Fermi energy level (*μc*) of graphene. This nicely clarifies the essential point that the broad absorption peak is tunable by varying the chemical potential of graphene. In other words, get to choose the part of the light spectrum to which the material most effectively absorbs, when the chemical potential is changed from *0.9 eV* to *0.1 eV* the absorption is reduced to 50%. In other words, the metamaterial with VO_2_ in metallic state exhibits a broadband absorption modulation with the efficiency above 90% as the shown in Figure 7a. The wideband absorption characteristics of the metamaterial are primarily governed by the phase transition properties of VO_2_. VO_2_ exhibits a significant change in conductivity (several orders of magnitude) during its insulator-to-metal transition (IMT), which directly modulates the electromagnetic response of the structure. This strong dependence arises because the IMT of VO_2_ alters the effective impedance of the metamaterial, enabling dynamic tuning of the absorption spectrum across a wide frequency range. In contrast, graphene’s chemical potential, while influential in fine-tuning the absorption properties, does not induce such a dramatic change in the overall electromagnetic response.

Graphene’s chemical potential primarily affects its surface conductivity, which influences the absorption characteristics at specific resonant frequencies. However, in the context of wideband absorption, the effect of graphene’s chemical potential is relatively subtle compared to the large-scale changes induced by VO_2_’s conductivity. This is because the wideband absorption mechanism relies more on the collective response of the metamaterial structure, which is dominated by the VO_2_ phase transition. While graphene contributes to the overall performance, its role is secondary in this particular design.

The chemical potential or Fermi level of the graphene layer can be adjusted in practice using methods such as electrostatic gating, chemical doping, or optical excitation, as demonstrated in studies like [41], to enhance performance in applications such as cancer detection and terahertz devices. We propose the use of an ion-gel layer to facilitate doping. Ion-gel gating is a practical method for applying an electric field to isolated graphene structures, as it allows for efficient control of the Fermi level without requiring direct electrical connections. However, this approach may introduce fabrication challenges, such as: Uniform Coating: Ensuring a uniform and thin ion-gel layer over the isolated graphene elements can be difficult, potentially leading to uneven doping. Compatibility: The ion-gel material must be compatible with the graphene and substrate to avoid degradation or contamination during fabrication. Stability: Long-term stability of the ion-gel layer under operational conditions (e.g., temperature, humidity) may be a concern, requiring encapsulation or protective measures. These issues can be mitigated through careful material selection, optimized fabrication techniques, and post-processing treatments

In this excerpt different levels of the Fermi energy of graphene are considered while VO_2_ is present in its insulating state Figure 7b. A high-frequency blue shift occurs with changes in Fermi energy. When the absorption peak shifts to higher frequencies, it is referred to as a blue shift. The good performance of multi-band seems to appear, e.g., at a Fermi energy level of 0.9 eV. This probably means multiple absorption peaks at various frequencies, which might be desired by some applications. In Figure 7b, the similarity in the absorption spectra at chemical potentials of 0.1 eV and 0.9 eV arises because, in the insulating state of VO_2_, the dielectric properties of VO_2_ dominate the overall optical response of the metamaterial, effectively masking the subtle variations in graphene’s conductivity induced by changes in chemical potential. While it is true that graphene’s conductivity changes smoothly with chemical potential due to its linear band dispersion near the Dirac point, the influence of these changes on the absorption spectrum is significantly attenuated when VO_2_ is in its insulating phase, as the large dielectric contrast of VO_2_ overshadows the relatively minor contributions from graphene’s conductivity. This dominance of VO_2_’s dielectric properties results in the observed similarity of the spectra at 0.1 eV and 0.9 eV, despite the expected shift in graphene’s Fermi level. However, we acknowledge that the spectra at intermediate chemical potentials (0.3, 0.5, and 0.7 eV) exhibit a gradual shift toward higher energies, which aligns with the reviewer’s expectation of a smooth adjustment. This behavior can be attributed to the interplay between graphene’s conductivity and VO_2_’s dielectric properties, where the influence of graphene becomes more pronounced at intermediate chemical potentials but is still insufficient to significantly alter the spectrum at the extremes (0.1 eV and 0.9 eV). General speaking, the spectrum features confirmation suggested that the light absorb properties of this metamaterials design can be effectively influenced by the graphene/VO_2_ coordination. The researchers can tune adiabatically and achieve highly efficient tunable broadband light absorption by controlling the Fermi energy level of graphene and the state of VO_2_.

**Figure 8:**
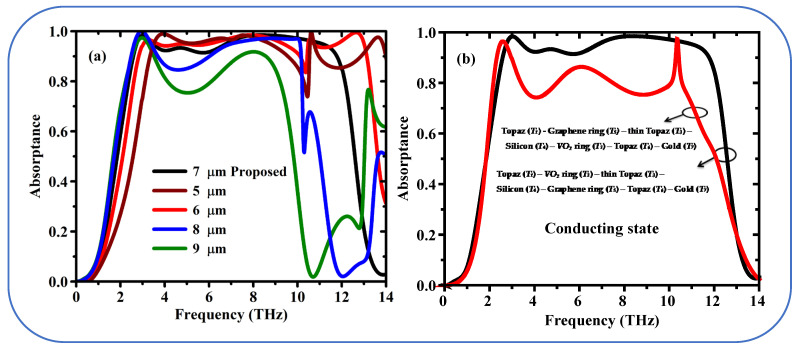
(**a**) Numerical absorption results for the various thickness of the topaz layer (*T*_1_ *Topaz*) when the VO_2_ in conducting state, and (**b**) absorption spectrum when VO_2_ in conducting state for the two different design from the top to bottom: Topaz (*T*_1_)—VO_2_ ring (*T*_2_)—thin Topaz (*T*_3_)—Silicon (*T*_4_)—Graphene ring (*T*_5_)—Topaz (*T*_6_)—Gold (*T*_7_), (proposed structure) Black line and Topaz (*T*_1_)—Graphene ring (*T*_5_)—thin Topaz (*T*_3_)—Silicon (*T*_4_)—VO_2_ ring (*T*_2_)—Topaz (*T*_6_)—Gold (*T*_7_) (Red Line).

Figure 8a illustrates the absorption dependence on the thickness variation of the Topaz layer (*T*_1_). In this graphene/VO_2_ metamaterial design, optimizing the thickness *T*_1_ of the Topaz layer is critical for achieving ultra-broadband absorption. The proposed design achieves an absorption band from 2.52 THz to 11.62 THz at an optimal *T*_1_ thickness of 7 μm. For thicknesses less than 7 μm, the absorption bandwidth narrows, and a noticeable decrease in absorption to ~80% are observed around 10.6 THz for thicknesses of 5 and 6 μm. Conversely, when the thickness is increased to 8 or 9 μm, the impedance matching condition is disrupted, leading to a significant drop in absorption across the entire frequency range. This behavior indicates that the blackbody-like response is eliminated, and the broadband absorption performance deteriorates. These results highlight the importance of precise tuning of the Topaz layer thickness to achieve the desired metamaterial functionality within the presented structure.

Figure 8b illustrates the absorbance spectra of two different metamaterial designs in the VO_2_ conducting phase. The black line corresponds to the schematic configuration (Au-T_1_-VO_2_-Si-Graphene-T_2_), with ultra-broadband modulated absorption for the proposed system. And conversely for the absorption spectrum when the roles of Graphene and VO_2_ exchanged, as follows by the red curve. The absorption is therefore strongly diminished until below 80% at a wider frequency range. The comparison shows the significance of the layer order (VO_2_ on the top of graphene) for the designed ultra-broadband absorption properties.

**Figure 9:**
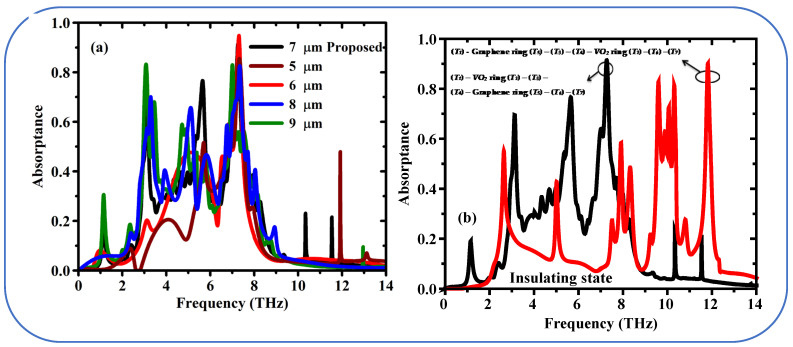
(**a**) Simulated absorption spectrum for the proposed metamaterial design when VO_2_ in insulating state (**a**) various thickness of the topaz layer (*T*_1_ Topaz), and (**b**) exchanged graphene and VO_2_ layer (Red Line).

Figure 9a studies the effect of topaz layer thickness (*T*_1_) on the absorption property of metamaterial in the VO_2_ insulating state. The designed parameter is able to support tri-band absorption feature with three distinct resonance peaks at 7.24 THz, 5.65 THz and 3.12 THz. This tailored thickness is important for achieving the intended multi-band behavior. With the thickness equal to 5 and 6 micron can see only one absorption peak and its position is 7 THz. On the other hand, a thickness of 8 and 9 μm maintains the tri-band response, but the overall absorption strength is significantly reduced. This also confirms the fine tuning of the topaz layer for implementing the multi-band feature in the proposed design.

Figure 9b shows the absorption spectrum of the two designs (black and red lines). The proposed structure (Au-T_1_-Gr-Si-Tthin-VO_2_-T_2_) again displays the multi-band response with three clear peaks of >80% absorption in the black line. The red solid line stands for a design change where VO_2_ and graphene are swapped (Au-T_1_-VO_2_-Si-T_thin_-Gr-T_2_). This change modifies the absorption profile leading to multi peaks were is only the three first peaks exceed 80% absorption. In order to achieve the desired strongly absorbing multi-band response in the proposed design, the comparison agrees with the role of the layer order is specific.

**Figure 10:**
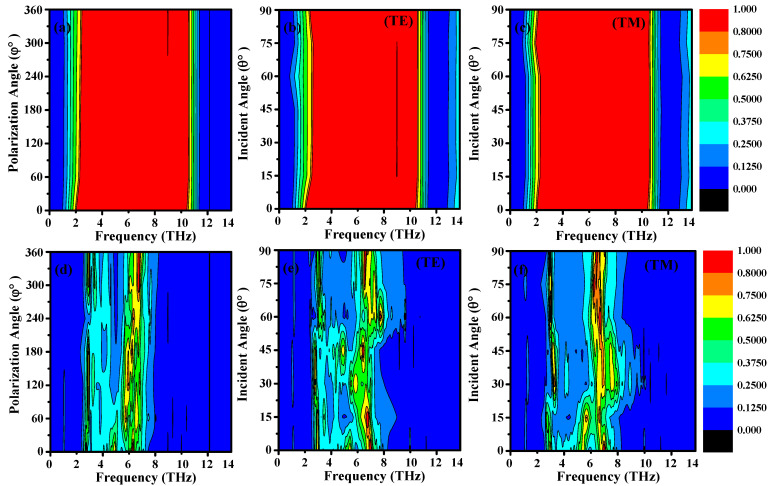
Numerical absorption color map under the condition of fermi energy level of 0.9 eV for the polarization angle, incident angle TE and TM mode for the broadband (**a**–**c**) and multi-band (**d**–**f**).

Figure 10a Details the polarization-independent broadband absorption responses of the designed configuration when the VO_2_ is in the conducting state. This plots a color map of absorption from 0 to 14 THz for several different incident angles (0 to 360 degrees in 60 degree steps), We see that the results are not very sensitive to polarization angle. Even though there is a gradual red shift (an increasingly lower wavelength with increasing angle) and the observation is made from 0 to 360 degrees, the absorption stills holding high values, this properties achieved due to symmetry of the metamaterials design, which is important for THz absorber. Nevertheless, this results in the drop of the absorption at one specific frequency (about 10.36 THz) when the polarization angle is elevated. This implies that while the overall design has polarization-independence, there may exist certain bands where the polarization effects begin to manifest. The black line in the red region represents a dip in absorption at specific incident angles and frequencies. This occurs due to the destructive interference of electromagnetic waves at the metamaterial surface, which is influenced by the angle of incidence. At certain angles, the phase matching condition for perfect absorption is not met, leading to a reduction in absorption efficiency. This phenomenon is inherent to the design and behavior of the metamaterial structure, particularly when operating under broad-angle incidence.

The virtualized and multi-performance analysis renders the criticality of the incident angle in the broad-band resonance absorption properties of the proposed metamaterial design in VO_2_ conducting states, as illustrated in Figure 10b,c. For the TE mode in Figure 10b, the black line in the red region similarly indicates a reduction in absorption at specific angles and frequencies. This is attributed to the polarization-dependent response of the metamaterial. In the TE mode, the electric field is perpendicular to the plane of incidence, and at certain angles, the coupling between the incident wave and the metamaterial structure weakens, resulting in lower absorption. This effect is more pronounced in the TE mode due to the anisotropic nature of the structure and the interaction of the electric field with the graphene-VO_2_ layers. In both cases, the black lines are a result of the complex interplay between the incident angle, frequency, and the metamaterial’s design. These effects are consistent with the theoretical expectations for such tunable and switchable absorbers

The color maps in Figure 10b,c, depict an analysis of the resonance absorptivity characteristics for a wide range of frequencies ranging between 0 to 14 THz and the incident angle flowing between 0 to 90 degrees in steps of 15 degrees for the two incident modes of TE and TM. As the degree of incident shifted from 0 to 90 degrees, a noticeable effect was evidenced that the absorptivity characteristics became not highly sensitive to the angles of incidence. It is noteworthy across all frequency bands. In fact increasing the angle of incidence would decrease the absorption effects at the resonant positions of 5.33 THz and 10.36 THz. This highlights implies the highly phenomena that need consideration during the design of metamaterials that require more structural consideration when fixing the design and it is important for real world applications.

Figure 10d Color map of the absorption of the metamaterial in VO_2_ insulating state over the characterization bandwidth, 0–14 THz, for the multiband response for a series of angles of polarization (from 0° to 360°, with 60°) are shown in Figure 10d, color map showing the absorption response with very little dependence on the polarization angle change (0° to 360°). Strikingly, both the first and second resonance peaks show significant red shifts with increasing angle. Figure 10e,f show color maps with the simulation absorption spectra of the metamaterial with the VO_2_ insulating state in the multiband response (0–14 THz). The maps depict both the TE and TM wave incident modes from 0 to 90 degrees along with the incidence angles at 15-degree intervals. The results obtained display a strong dependence of absorption with the incident angle for both TE and TM modes. When the angle of incidence changes gradually from 0 to 90 (deg), the frequency-dependent absorption profile shows significant modulation of amplitude, indicating that angles of incidence should be considered for design of practical devices based on this metamaterial. The incidence angle of incoming light significantly affects the optical properties of metamaterials, and thus involving into the design of metamaterials. Polarization-dependent responses: Many metamaterials exhibit polarization-dependent behavior, meaning their absorption, reflection, or transmission properties can vary depending on the orientation of the electric field in the incident light wave.

## 4. Evaluating Sensing Performance

**Figure 11:**
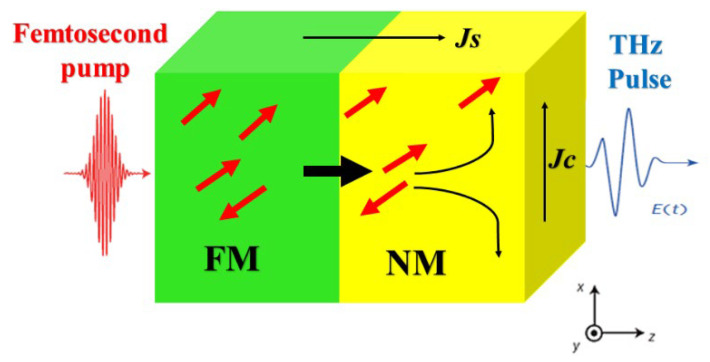
Illustrates Metallic spintronic THz emitter principle of operation.

This section outlines a framework designed to detect two types of cancerous cells (Cervical and Breast). The proposed detection and sensing system is designed for narrowband terahertz (THz) applications, operating within the frequency range supported by VNA-based spectroscopy (up to approximately 1 THz) which is employs a one-port reflection coefficient measurement method, where an antenna functions as a microwave sensor. The test sample is prepared by sandwiching it between coverslip layers and placing it directly on the antenna’s radiating aperture. The antenna is linked to a vector network analyzer (VNA), which is then connected to a computer to track variations in the *S*_11_ response (Δ*S*_11_) and resonance frequency shift (Δ*f*_0_) when normal and cancerous cells are placed on the aperture. A key benefit of this system is that the human body remains in the far-field region of the antenna’s radiation pattern, reducing exposure to electromagnetic waves. As a result, the specific absorption rate (SAR) is anticipated to stay within the safety limit of 1.6 W/kg. For broadband THz measurements (up to 10 THz), alternative techniques such as optically pumped spintronic materials (THz-TDS) are recommended, as demonstrated in recent studies [42], the basic principle of spintronic terahertz is illustrated in in Figure 11 emitter with a ferromagnetic (FM) bilayer and non-ferromagnetic (NM) metal thin films [43]. The FM layer is antiparallel to the y axis, with the in-plane magnetization. An incident femtosecond a laser pulse excites electrons in the metals to states above the Fermi energy determining their band velocity and scattering rate. Due to the different transport properties of the FM and NM layers, sending a net current along the z axis. In addition, because the product of the density, band velocity and lifetime of spin-up (majority) electrons, in FM metals like (Fe, Co and Ni) are remarkably more than on the spin-down (majority) electrons [44], the z current is highly spin-polarized [45]. On entering the NM layer, spin-orbit coupling deflects spin-up and spin-down electrons in opposite directions [44] by a mean near-angle γ (Figure 11). This (ISHE) of inverse spin-Hall effect converts the longitudinal (z-directed) spin current density *j_s_* into an ultrafast transverse *j_c_* = *γj_s_*, (x-directed) hence charge current density serving as a terahertz radiation source.

**Figure 12:**
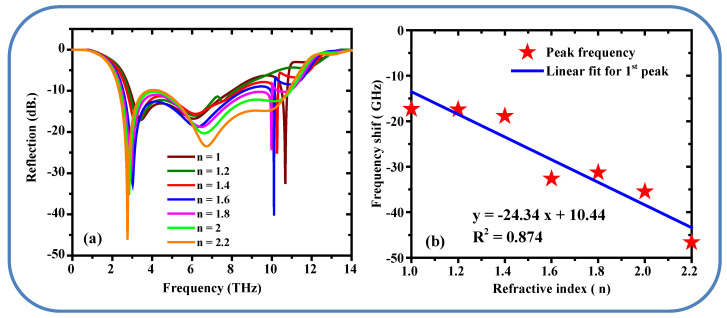
(**a**) Effect of the refractive index (n) on the S_11_ response. (**b**) Relationship between the refractive index (n) and the resonance frequency shift.

The proposed Metamaterials structure was evaluated within the previously mentioned detection system. To assess and improve the absorber’s sensitivity, one analyte sections inside the metamaterials structure was designed and positioned on the antenna’s radiating aperture, as depicted in Figure 11.The *S*_11_ results, shown in Figure 12a, changing the refractive index of the surrounding from 1 to 2.2 in steps 0.2, as it seen from the figure n increased the resonance frequency *f*_0_ shifts to the lower frequency, This shift can be used to determine the sensitivity of the metamaterials absorber [46]. A red shift (shift to lower frequencies) typically occurs when the effective refractive index of the metamaterial increases. This can be caused by: Increased coupling between the metamaterial structure and the incident electromagnetic waves at lower frequencies. Changes in the effective permittivity or permeability of the material, which are more pronounced at lower frequencies due to the longer wavelength and stronger interaction with the material’s intrinsic properties. Structural or material modifications that enhance the inductive or capacitive effects, leading to a lower resonant frequency.

Blue Shift at High Frequencies (>6 THz): A blue shift (shift to higher frequencies). This can be due to: Reduced coupling between the metamaterial and the incident waves at higher frequencies, where the wavelength is shorter and the interaction is weaker. Changes in the material’s dispersion properties, which can cause the high-frequency resonances to shift upward. In Figure 12a the refractive indices for both peaks are consistent at around 2.98, with the first peak at 4 THz and the second peak at 11 THz. Furthermore, the reflection spectra for normal and cancer cells now show nearly identical behavior for both peaks, indicating minimal differences in their optical properties at these frequencies.

Figure 12b presented the linear shift and fitting curve for the proposed metamaterials design when adding alayte layer to the structure, the values are as follows: Adj.R-Square = 0.874, intersection is 10.34, slop is −24.34, it means the sensitivity of the proposed design for the first peak is 0.2489 THz/RIU. From the graph indicating a robust linear relationship between changes in n and frequency shifts. In future work, we will incorporate a biocompatible coating (e.g., silicon dioxide, SiO_2_, or polydimethylsiloxane, PDMS) on the gold (Au) layer in the proposed design. These materials are widely used in biosensing due to their non-toxicity, chemical stability, and excellent biocompatibility. This surface pretreatment will ensure that the metamaterial is safe for interaction with living cells while maintaining its terahertz absorption and sensing performance [47].

**Figure 13:**
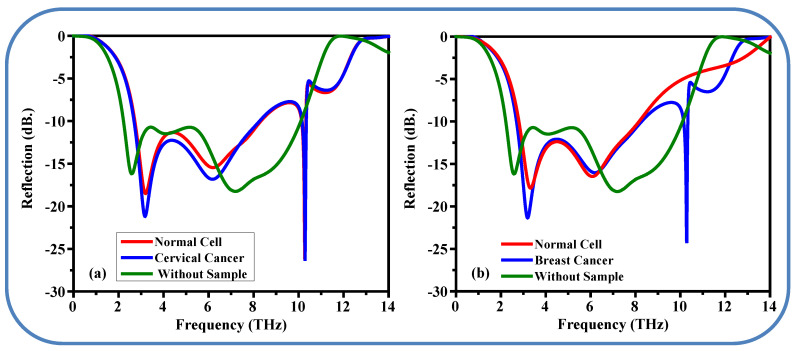
Simulated S_11_ response of the proposed metamaterials absorber for healthy tissue, without sample and cancer cells. (**a**) Cervical cancer and (**b**) breast cancer.

The proposed metamaterial absorber is also employed for diagnosing cancerous cells, as illustrated in Figure 13. To ensure accurate and error-free results, the sample is sandwiched between two coverslip layers. The testing process involves placing both normal and cancerous cell samples (cervical, Figure 13a, and breast, Figure 13b) between the coverslips and positioning them on the radiating aperture. The identification of cancerous cells is based on the difference in the refractive index (n) between healthy and cancerous cells, allowing for precise characterization using Δ*f*_0_ and Δ*S*_11_ values. A simplified model of the frequency dependence of n, as described in [4], is utilized to characterize the sample materials. The refractive index (n) for normal cells is approximately 1.368, while for cervical cancer cells, it is 1.392; for breast cancer and normal cells, the values are 1.399 and 1.385, respectively [36].

Figure 13a shows the reflection coefficient for healthy cervical tissue, cervical cancer cells, and the case when no sample is placed between the two coverslips. As seen in the graph, the reflection coefficient for healthy cervical tissue is −18.67 at a frequency of 3.19 THz. When a cervical cancer cell is placed between the two coverslips, the reflection coefficient decreases from −18.67 to −21.39 at the resonance frequency of 3.18 THz.

In the sensing performance section, another type of breast cancer is used to evaluate the sensitivity of the proposed metamaterial, as shown in Figure 13b. For normal breast cells, the S_11_ is −18.21 at a frequency of 3.32 THz, while for breast cancer cells, the frequency shifts lower to 3.19 THz, and the S_11_ decreases to −21.48. In general, cancer cells contain more water, resulting in greater absorption of electromagnetic waves compared to normal cells, and thus reflect less electromagnetic energy. Figure 13 demonstrates that the proposed metamaterial absorber is sensitive and capable of detecting different types of cancer.

In Table 2, the comparison between the proposed metamaterial design and the others available in the literature is shown. We concentrate the comparison on the most relevant aspects, as indicated by the absorption band, material of choice, bandwidth, absorption rate, and year of publication. Our proposed design performs better than the other devices listed in the table from the perspective of the characterization of broadband absorption. The structure is capable of achieving ultra-broadband and high absorption properties, as demonstrated by portion of high absorption and superior bandwidth shown in the results.

## 5. Fabrication Techniques and Future Work

The proposed 7-layer structure, consisting of alternating graphene, VO_2_, and dielectric layers, is indeed complex but achievable with current nanofabrication technologies. Techniques such as chemical vapor deposition (CVD) for graphene growth, pulsed laser deposition (PLD) or sputtering for VO_2_ deposition, and spin-coating or atomic layer deposition (ALD) for dielectric spacers are well-established and have been successfully used to fabricate similar multi-layer metamaterial structures. Gold is typically deposited using thermal evaporation or sputtering, both of which are standard techniques for creating thin, uniform metal films. While the multi-layer structure is complex, layer-by-layer fabrication and alignment can be achieved using advanced nanofabrication tools such as electron-beam lithography (EBL) or focused ion beam (FIB) milling. Additionally, techniques like transfer printing and layer stacking have been successfully employed in similar multi-layer metasurface designs. We acknowledge that experimental validation is essential to demonstrate the practicality of the proposed structure. In future work, we plan to collaborate with experimental groups to fabricate and characterize the proposed metamaterial absorber. Preliminary simulations and theoretical analyses, as presented in this manuscript, are a crucial first step in guiding the design and optimization of the structure before experimental realization.

## 6. Conclusions

This study presents a terahertz metamaterial absorber with switchable and tunable absorption properties. The proposed design utilizes a layered structure combining graphene and VO_2_, allowing for control over the absorption response by manipulating the phase state of VO_2_. In the insulating state of VO_2_, the design exhibits a triple-band response with distinct absorption peaks at 3.12 THz, 5.65 THz, and 7.24 THz. However, when VO_2_ transitions to its conducting state, the design demonstrates an exceptionally broad absorption band ranging from 2.52 THz to 11.62 THz.

The absorption mechanisms of the proposed terahertz metamaterial (THz MTM) in both states are analyzed through electric field distributions at the resonance frequencies. This work investigates the influence of material properties on absorption by analyzing the effects of VO_2_ conductivity, loss tangent, and graphene’s chemical potential. Additionally, the influence of the Topaz layer thickness on absorption is numerically presented. Furthermore, the study investigates the absorption behavior of VO_2_ by analyzing different states and layer configurations. The variations in absorption due to polarization and incident angle are also presented.

The proposed design is utilized for detecting cervical and breast cancer, achieving a sensitivity of approximately 0.2434 THz/RIU. The proposed metamaterial design holds significant promise for real-world applications. Importantly, its reconfigurability offers the potential to tailor absorption properties across an even broader terahertz frequency range. This versatility makes it suitable for advanced devices such as filters, modulators, and perfect absorbers.

## Figures and Tables

**Table 1:** Defined Parameters of the Proposed Metamaterial Unit Cell (μm).

**Parameters**	*P*	*L*	*M*	*N*	*T*_1_	*T*_2_	*T*_3_	*T*_4_	*T*_5_
**Value**	20	20	16	13	6	0.2	0.2	0.2	0.2
**Parameters**	*T*_6_	*T_7_*	*g*	*l*_1_	*l*_2_	*l*_3_	*l*_4_	*r*_1_	*r*_2_
**Value**	7	0.2	1	4.5	2.04	3.5	1	2	5

**Table 2:** Comparison of Suggested metamaterials structure with previous study.

Ref.	Materials Selection	Band-Width (THz)	Absorption Band	Absorption Rate	Year Publication
[26]	Graphene + VO_2_	1.06–2.58	Broad–band & Multi band (3 Peaks)	>90%	2020
[27]	Graphene + VO_2_	2.5–4.30	Broad –band	>90%	2021
[29]	*VO2*	1.31–4.72, 8.19–11.44	Dual-band	>90%	2023
[30]	Graphene + VO_2_	1.05–1.6	Broad-band	>90%	2020
[31]	Graphene + VO_2_	0.94–4.31	Broad–band & Dual band (2 Peaks)	>90%	2023
[33]	Graphene + VO_2_	5.1–7.3	Broad–band	>90%	2022
[34]	Graphene + VO_2_	1–3.55	Broad–band & Dual band (2 Peaks)	>90%	2023
[35]	Graphene + VO_2_	2–5	Broad–band & Single band	>90%	2024
This work	Graphene + VO_2_	2.52–11.62	Ultra-Broad–band & Multi band (3 Peaks)	>90%	------

## Data Availability

The original contributions presented in the study are included in the article, further inquiries can be directed to the corresponding author.

## References

[1] Takida Y., Nawata K., Minamide H. (2021). Security screening system based on terahertz-wave spectroscopic gas detection. Opt. Express.

[2] Xu K., Arbab M.H. (2024). Terahertz polarimetric imaging of biological tissue: Monte Carlo modeling of signal contrast mechanisms due to Mie scattering. Biomed. Opt. Express.

[3] Jiang W., Zhou Q., He J., Habibi M.A., Melnyk S., El-Absi M., Han B., Di Renzo M., Schotten H.D., Luo F.-L. (2024). Terahertz Communications and Sensing for 6G and Beyond: A Comprehensive Review. IEEE Commun. Surv. Tutor..

[4] Hamza M.N., Abdulkarim Y.I., Saeed S.R., Hamad M.A., Muhammadsharif F.F., Bakır M., Appasani B., Haxha S. (2024). A Very Compact Metamaterial-Based Triple-Band Sensor in Terahertz Spectrum as a Perfect Absorber for Human Blood Cancer Diagnostics. Plasmonics.

[5] Mukherjee P., Banerjee S., Pahadsingh S., Bhowmik W., Appasani B., Abdulkarim Y.I. (2023). Refractive index sensor based on terahertz epsilonnegative metamaterial absorber for cancerous cell detection. J. Optoelectron. Adv. Mater..

[6] Hamza M.N., Abdulkarim Y.I., Saeed S.R., Altıntaş O., Mahmud R.H., Appasani B., Ravariu C. (2022). Low-cost antenna-array-based metamaterials for non-invasive early-stage breast tumor detection in the human body. Biosensors.

[7] Ali S.H., Abdulkarim Y.I., Mohammed H.O. (2024). Flexible wearable antenna incorporating metasurface for WBAN applications. Passer J. Basic Appl. Sci..

[8] Wu R., Dong J., Wang M., Abdulkarim Y.I. (2023). Wearable antenna sensor based on bandwidth-enhanced metasurface for elderly fall assistance detection. Measurement.

[9] Hevin A.M., Abdulkarim Y.I., Abdoul P.A., Dong J. (2023). Textile and metasurface integrated wide-band wearable antenna for wireless body area network applications. AEU-Int. J. Electron. Commun..

[10] Zerrad F.-E., Taouzari M., Makroum E.M., Islam M.T., Özkaner V., Abdulkarim Y.I., Karaaslan M. (2022). Multilayered metamaterials array antenna based on artificial magnetic conductor’s structure for the application diagnostic breast cancer detection with microwave imaging. Med. Eng. Phys..

[11] Das P., Madhav B.T.P. (2024). Convoluted I-Shaped Metamaterial on Rigid and Flexible Substrates for Electromagnetic Cloaking. J. Electron. Mater..

[12] Zhang Q., Hu G., Rudykh S. (2024). Magnetoactive asymmetric mechanical metamaterial for tunable elastic cloaking. Int. J. Solids Struct..

[13] Banerjee S., Dutta P., Basu S., Mishra S.K., Appasani B., Nanda S., Abdulkarim Y.I., Muhammadsharif F.F., Dong J., Jha A.V. (2022). A new design of a terahertz metamaterial absorber for gas sensing applications. Symmetry.

[14] Abdulkarim Y.I., Dalgaç Ş., Alkurt F.O., Muhammadsharif F.F., Awl H.N., Saeed S.R., Altıntaş O., Li C., Bakır M., Karaaslan M. (2021). Utilization of a triple hexagonal split ring resonator (SRR) based metamaterial sensor for the improved detection of fuel adulteration. J. Mater. Sci. Mater. Electron..

[15] Abdulkarim Y.I., Alkurt F.Ö., Bakır M., Awl H.N., Muhammadsharif F.F., Karaaslan M., Appasani B., Al-Badri K.S.L., Zhu Y., Dong J. (2022). A polarization-insensitive triple-band perfect metamaterial absorber incorporating ZnSe for terahertz sensing. J. Opt..

[16] Mazare A.G., Abdulk Y.I., Karim A.S., Bakır M., Taouzari M., Muhammadsharif F.F., Appasani B., Altıntaş O., Karaaslan M., Bizon N. (2022). Enhanced Sensing Capacity of Terahertz Triple-Band Metamaterials Absorber Based on Pythagorean Fractal Geometry. Materials.

[17] Ali H.O., Al-Hindawi A.M., Abdulkarim Y.I., Karaaslan M. (2022). New compact six-band metamaterial absorber based on Closed Circular Ring Resonator (CCRR) for Radar applications. Opt. Commun..

[18] Liu M., Plum E., Li H., Li S., Xu Q., Zhang X., Zhang C., Zou C., Jin B., Han J. (2021). Temperature-Controlled Optical Activity and Negative Refractive Index. Adv. Funct. Mater..

[19] Liu M., Plum E., Li H., Duan S., Li S., Xu Q., Zhang X., Zhang C., Zou C., Jin B. (2020). Switchable Chiral Mirrors. Adv. Opt. Mater..

[20] Liu M., Xu Q., Chen X., Plum E., Li H., Zhang X., Zhang C., Zou C., Han J., Zhang W. (2019). Temperature-Controlled Asymmetric Transmission of Electromagnetic Waves. Sci. Rep..

[21] Rengasamy S., Natarajan R., Srinivasan V.K. (2023). Miniaturized Multi-Spectral Perfect Metamaterial Absorber for THz Sensing, Imaging and Spectroscopic Applications. Plasmonics.

[22] Rani N., Bohre A.K., Bhattacharya A. (2023). VO2 based multi-functional ultra-wideband terahertz meta-absorber for EMI shielding application. Smart Sci..

[23] Hao S., Wang J., Fanayev I., Khakhomov S., Li J. (2023). Hyperbolic metamaterial structures based on graphene for THz super-resolution imaging applications. Opt. Mater. Express.

[24] Ma S., Wen S., Mi X., Zhao H. (2023). Terahertz optical modulator and highly sensitive terahertz sensor governed by bound states in the continuum in graphene-dielectric hybrid metamaterial. Opt. Commun..

[25] Zamzam P., Rezaei P., Abdulkarim Y.I., Daraei O.M. (2023). Graphene-based polarization-insensitive metamaterials with perfect absorption for terahertz biosensing applications: Analytical approach. Opt. Laser Technol..

[26] Zhu H., Zhang Y., Ye L., Li Y., Xu Y., Xu R., Xu H., Xu M. (2020). Switchable and tunable terahertz metamaterial absorber with broadband and multi-band absorption. Opt. Express.

[27] Liu Y., Huang R., Ouyang Z. (2021). Terahertz absorber with dynamically switchable dual-broadband based on a hybrid metamaterial with vanadium dioxide and graphene. Opt. Express.

[28] Liu M., Wei R., Taplin J., Zhang W. (2023). Terahertz Metasurfaces Exploiting the Phase Transition of Vanadium Dioxide. Materials.

[29] Ri K.-J., Ri C.-H. (2023). Tunable dual-broadband terahertz metamaterial absorber based on a simple design of slotted VO2 resonator. Opt. Commun..

[30] Wang T., Zhang Y., Zhang H., Cao M. (2020). Dual-controlled switchable broadband terahertz absorber based on a graphene-vanadium dioxide metamaterial. Opt. Mater. Express.

[31] Song C., Wang J., Zhang B., Qu Z., Jing H., Kang J., Hao J., Duan J. (2023). Dual-band/ultra-broadband switchable terahertz metamaterial absorber based on vanadium dioxide and graphene. Opt. Commun..

[32] Zhang P., Chen G., Hou Z., Zhang Y., Shen J., Li C., Zhao M., Gao Z., Li Z., Tang T. (2022). Ultra-Broadband Tunable Terahertz Metamaterial Absorber Based on Double-Layer Vanadium Dioxide Square Ring Arrays. Micromachines.

[33] Zhuo S., Liu Z., Zhou F., Qin Y., Luo X., Ji C., Yang G., Yang R., Xie Y. (2022). THz broadband and dual-channel perfect absorbers based on patterned graphene and vanadium dioxide metamaterials. Opt. Express.

[34] Hu D., Jia N., Zhu Q. (2023). Switchable dual-broadband to single-broadband terahertz absorber based on hybrid graphene and vanadium dioxide metamaterials. Phys. Scr..

[35] Hossain A.B.M.A., Khaleque A. (2024). Multi-functional and actively tunable terahertz metamaterial absorber based on graphene and vanadium dioxide composite structure. Opt. Contin..

[36] Jabin A., Ahmed K., Rana J., Paul B.K., Islam M., Vigneswaran D., Uddin M.S. (2019). Surface Plasmon Resonance Based Titanium Coated Biosensor for Cancer Cell Detection. IEEE Photonics J..

[37] Ding Z., Su W., Wu H., Li W., Zhou Y., Ye L., Yao H. (2023). Ultra-broadband tunable terahertz absorber based on graphene metasurface with multi-square rings. Mater. Sci. Semicond. Process..

[38] Zhou Z., Song Z. (2022). Terahertz mode switching of spin reflection and vortex beams based on graphene metasurfaces. Opt. Laser Technol..

[39] Zhang Y., Li T., Chen Q., Zhang H., O’Hara J.F., Abele E., Taylor A.J., Chen H.-T., Azad A.K. (2015). Independently tunable dual-band perfect absorber based on graphene at mid-infrared frequencies. Sci. Rep..

[40] Wang D., Cui X., Liu D., Zou X., Wang G.-M., Zheng B., Cai T. (2024). Multi-Characteristic Integrated Ultra-Wideband Frequency Selective Rasorber. Prog. Electromagn. Res..

[41] Liu B., Peng Y., Hao Y., Zhu Y., Chang S., Zhuang S. (2024). Ultra-wideband terahertz fingerprint enhancement sensing and inversion model supported by single-pixel reconfigurable graphene metasurface. PhotoniX.

[42] Seifert T., Jaiswal S., Martens U., Hannegan J., Braun L., Maldonado P., Freimuth F., Kronenberg A., Henrizi J., Radu I. (2016). Efficient metallic spintronic emitters of ultrabroadband terahertz radiation. Nat. Photonics.

[43] Kampfrath T., Battiato M., Maldonado P., Eilers G., Nötzold J., Mährlein S., Zbarsky V., Freimuth F., Mokrousov Y., Blügel S. (2013). Terahertz spin current pulses controlled by magnetic heterostructures. Nat. Nanotechnol..

[44] Jin Z., Tkach A., Casper F., Spetter V., Grimm H., Thomas A., Kampfrath T., Bonn M., Kläui M., Turchinovich D. (2015). Accessing the fundamentals of magnetotransport in metals with terahertz probes. Nat. Phys..

[45] Battiato M., Carva K., Oppeneer P.M. (2010). Superdiffusive spin transport as a mechanism of ultrafast demagnetization. Phys. Rev. Lett..

[46] Yao J., Ou J.-Y., Savinov V., Chen M.K., Kuo H.Y., Zheludev N.I., Tsai D.P. (2022). Plasmonic anapole metamaterial for refractive index sensing. PhotoniX.

[47] Beliaev L.Y., Lavrinenko A.V., Takayama O. (2024). Alternative Plasmonic Materials for Biochemical Sensing: A Review. Prog. Electromagn. Res..

